# Quantitative Visualization of Lanthanum Accumulation in Lanthanum Carbonate-Administered Human Stomach Tissues Using Mass Spectrometry Imaging

**DOI:** 10.5702/massspectrometry.A0086

**Published:** 2020-07-13

**Authors:** Shuichi Shimma, Yoshiki Makino, Kazuto Kojima, Takafumi Hirata

**Affiliations:** 1 Department of Biotechnology, Graduate School of Engineering, Osaka University, Suita, Osaka 565–0871, Japan; 2 Geochemical Research Center, The University of Tokyo, Hongo, Tokyo 113–0033, Japan; 3 Keisuikai Oka Hospital, Honjo, Saitama 367–0031, Japan

**Keywords:** LA-ICP-MS, mass spectrometry imaging, lanthanum, macrophage, toxicology

## Abstract

Platinum, a transition metal that is widely used in anti-cancer agents, also results in the development of nephropathy due to severe adverse reactions caused by platinum-induced nephrotoxicity. Reports on imaging with metals other than platinum remain are limited, even in preclinical studies. Furthermore, most of these are case reports, and the relationship between the distribution of the metal and clinical observations in human samples is not well understood. Here we report on visualizing lanthanum (^139^La), a component of Fosrenol, which is usually used for the treatment of hyperphosphatemia. Gastric inflammation, also known as hemorrhagic gastritis, is the main adverse event caused by Fosrenol. To conduct this study, ^139^La was visualized in gastric biopsy samples obtained from a patient using quantitative laser ablation-inductively coupled plasma-mass spectrometry (LA-ICP-MS). We also compared the distribution of ^139^La in tissue and histochemical results. The areas where ^139^La accumulated corresponded to the macrophage-positive areas observed in immunohistochemistry studies using an anti-CD68 antibody. In contrast, we observed a debris-like crystal morphology in hematoxylin and eosin staining tissues. The debris was also associated with ^139^La accumulation. The abnormal accumulation of ^139^La crystals caused the observed inflammation. This phenomenon was previously characterized, but this is the first report in which ^139^La distribution and histochemical results are compared using LA-ICP-MS.

## INTRODUCTION

Metal imaging using laser ablation-inductively coupled plasma-mass spectrometry (LA-ICP-MS) has recently become a popular method that is used in many research fields, including basic biology and toxicology. An important feature of LA-ICP-MS imaging is its ability to provide quantitative data, including a wide dynamic range because the metals and efficiently ionized, even on the tissue surfaces. Therefore, many reports concerning metal imaging using LA-ICP-MS have appeared, including those on the endogenous distribution of metals in animal and human tissues,^1–3)^ toxicological studies related to exogenous cadmium in rat fetuses,^4)^ effects of dibutyltin on placental and fetal toxicity in rats,^5)^ and lead poisoning *via* accumulation in the bones of water birds.^6)^

Additionally, toxicological studies of pharmaceuticals that contain metals are also important applications of LA-ICP-MS imaging.^7–11)^ Pharmaceutically relevant metals include alkali, alkaline earth, and transition metals and are used widely in drug development. Table 1 provides a summary of pharmaceuticals and diseases that have been investigated using LA-ICP-MS. As shown in Table 1, there are many reports on anti-cancer agents containing platinum, which inhibit DNA synthesis. A major adverse event of these platinum-based compounds includes nephrotoxicity, and almost all reports involved the visualization of the distribution of platinum inside kidney tissues.^11)^ As can be seen in Table 1, animal model studies predominate, and only a few reports on metal visualization using human clinical samples are available.

**Table 1:** Table 1. Summary of previously reported metal imaging in toxicological studies.

Authors	Disease	Pharmaceutical	Target metals	Significance
Moreno-Gordaliza *et al.*	Nephrotoxicity (Rats)	Cisplatin	Pt, Cu, and Zn	Cu and Zn distribution maps revealed significant displacement in cells by Pt
Bonta *et al.*	Pleural mesothelioma (Human)	Cytostatic drugs	Pt	Quantitative Pt imaging coupled with histological images of malignant areas (H&E staining and anti-CD34)
Egger *et al.*	Normal (Mice)	Cisplatin and KP1339	Pt and Ru	Quantitative ICP-MS imaging validated using conventional ICP-MS methods
Van Acker *et al.*	Nephrotoxicity (Cynomolgus monkeys)	Cisplatin	Pt	Comparison of high-resolution H&E staining results and the Pt distribution
Schreiber-Brynzak *et al.*	Tumor hypoxia model (cancer spheroids and xenografts)	Satraplatin and Pt-containing chemicals	Pt	Comparison and description of differences between hypoxia and non-hypoxic conditions

In this study, we visualized lanthanum (^139^La) using human gastric biopsy tissues using a quantitative imaging method.^12)^ Lanthanum, a component of the drug Fosrenol, is present in the form of lanthanum carbonate hydrate and is used in the treatment of hyperphosphatemia. The main adverse event of this drug is hemorrhagic gastritis. In this study, we compared the visualization of ^139^La based on ion images and histological observations in an attempt to elucidate hemorrhagic gastritis. Our findings revealed that a significant accumulation of ^139^La corresponded to macrophage-positive areas, as visualized using anti-CD68 antibodies.

## EXPERIMENTAL

### Chemicals

An anti-CD68 antibody, 1× Tris-buffered saline with Tween® 20, SignalStain® antibody diluent, SignalStain® Boost IHC detection reagents, and SignalStain® DAB substrate kits were purchased from Cell Signaling Technology (Danvers, MA, USA). Xylene, mounting medium, Mayer’s hematoxylin solution, and 1% Eosin Y solution were purchased from FUJIFILM Wako Pure Chemical Corporation (Osaka, Japan). Goat serum was purchased from Merck (Darmstadt, Germany).

### Specimen collection

The patient (a 66-year-old male) with hemorrhagic gastritis was subjected to gastric biopsies at the Oka hospital (Honjo, Saitama, Japan), which were collected for inspection. Written informed consent was obtained from the patient as a comprehensive agreement for future studies. The obtained tissues were stored as formalin-fixed, paraffin-embedded (FFPE) tissues. The samples were then anonymized during their analysis. For the LA-ICP-MS analysis, this study was approved by the Institutional Review Board of the University of Tokyo (17–29; Visualization of lanthanum distribution included in Fosrenol at inflammatory sites).

### Sectioning

The FFPE sample was sliced into 8 μm sections using a rotary microtome (RM2145, Leica, Wetzlar, Germany) and mounted on glass slides in a water bath at 40°C. The tissue slides were dried overnight at 37°C on a flattering plate (Sakura Finetek Japan, Tokyo, Japan). Serial sections were prepared and used for LA-ICP-MS imaging, immunohistochemistry, and hematoxylin and eosin (H&E) staining.

### Deparaffinization

Deparaffinization was performed by three cycles of washing with xylene at a temperature of approximately 25°C for 5 min. The sample for LA-ICP-MS analysis was directly dried to avoid the delocalization of the analytes. Samples for immunohistochemistry or H&E staining were further washed with 100% ethanol (10 min, twice), 95% ethanol (10 min, twice), and water (5 min, twice). After washing, the staining procedure was performed.

### Hematoxylin and eosin staining

The H&E staining protocol after deparaffinization was as follows: tissue slides were stained with Mayer’s hematoxylin solution for 1 min and washed with water (1 min, twice). The slides were then stained with 1% Eosin Y solution for 1 min and washed with water (1 min, twice). After staining, the samples were dehydrated in ethanol (1 min, three times), and permeation was performed with xylene (1 min, twice). The resulting stained tissues were then mounted with a coverslip using a mounting medium and dried overnight.

### Immunohistochemistry using an anti-CD68 antibody

CD68 is a transmembrane glycoprotein that is highly expressed on monocytes and macrophages. Herein, immunohistochemistry (IHC) for CD68 was performed to identify the inflammation sites. The anti-CD68 antibody (1 : 400, Cell Signaling Technology, 76437) staining protocol, including antigen retrieval, was obtained from Cell Signaling Technology.

### Visual inspection with a digital microscope

To observe the stained tissues, an all-in-one fluorescent microscope (BZ-X710, Keyence, Osaka, Japan) was utilized. To control the microscope and analyze the obtained pictures, BZ-X Viewer version 1.3.1.1 (Keyence) and BZ-X Analyzer version 1.3.1.1 (Keyence) were used.

### LA-ICP-MS imaging experiment

Laser ablation was performed using an in-house laser ablation system equipped with an IFRIT femtosecond laser (Cyber Laser, Tokyo, Japan). The laser ablation system was connected to iCAP-Qc (Thermo Fisher Scientific, Bremen, Germany). The operational settings of the LA-ICP-MS instrument were optimized to obtain the maximum intensity for ^139^La using a NIST SRM 610 glass standard material. The instrumentation and analytical conditions are summarized in Table 2.

**Table 2:** Table 2. Experimental parameters.

ICP-MS (Thermo Fisher Scientific iCAP-Qc)
RF power (W): 1,400
Cooling gas flow rate (L/min): 14
Auxiliary gas flow rate (L/min): 0.8
He carrier gas (L/min): 0.6
Ar make up gas (L/min): 1.00–1.05
Sampling depth: 5
LASER (In House Laser Ablation System)
Wavelength (nm): 266
Pulse width (fs): 190
Repetition frequency (Hz): 10
Spot size (μm): 15
Scan speed (μm/s): 10

### Image reconstruction and quantification

Image reconstruction was performed using the iQuant2 software.^13)^ For quantitative imaging, a previously published method calibrated by NIST SRM 610 was used.^12)^

## RESULTS AND DISCUSSION

### Quantification using NIST SRM610

**Figure 1:**
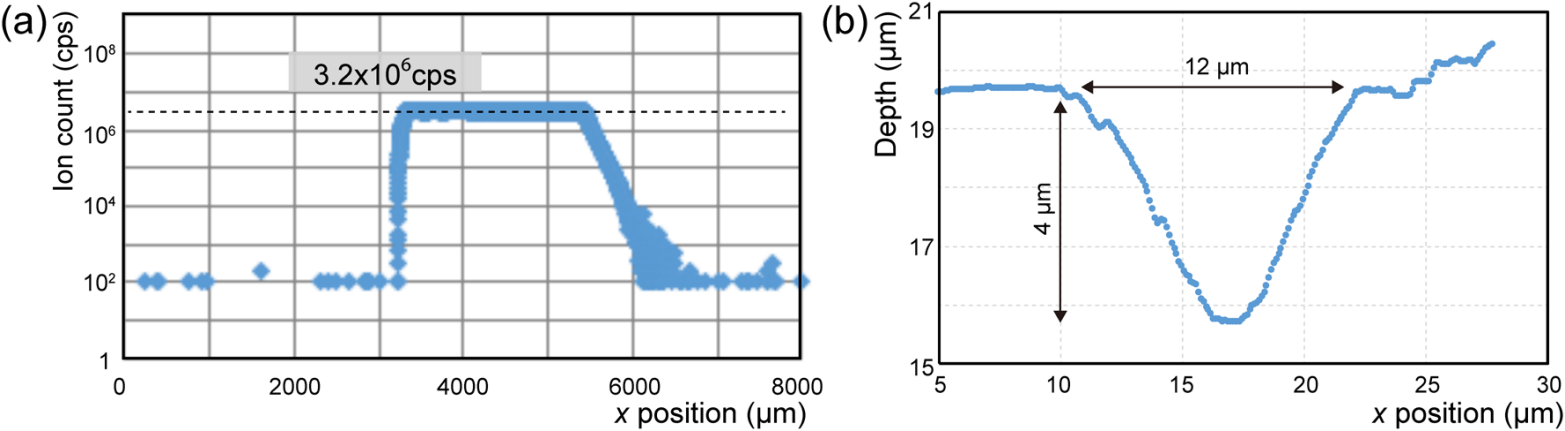
Fig. 1. Ion count of ^139^La and trace of ablation of the NIST SRM 610 trace element glass. (a) On the glass, the ion count of ^139^La plateaued at 3.2×10^6^ cps. (b) Trace of laser ablation on the glass. Under these experimental settings, the depth and diameter were determined to be 4 and 12 μm, respectively, by optical microscopy.

To convert the raw ion count into concentration in the human tissue sample, ^139^La detection was performed using the NIST SRM610 standard under the same conditions as were used for the tissues. The resulting ion count is shown in Fig. 1a. According to the SRM610 measurement, a plateau in ion intensity was observed on the glass, at a ^139^La count of 3.2×10^6^ cps. However, since the ablated volumes would differ between the tissue section and glass standard, a correction for the ablated volume difference was made. The trace of the laser ablation was examined by optical microscopy to estimate the ablated volume of the glass standard for comparison to the ablated volumes of the tissue sections with glass. The obtained diameter and depth values were found to be 12 and 4 μm, respectively, and an intensity value of 3.2×10^6^ cps was obtained from this spot. Using the measured volumes of the standard glass and tissue section, the signal intensity was corrected using the volume ratio based on the equation below, and quantitative values were obtained from the imaging results. 
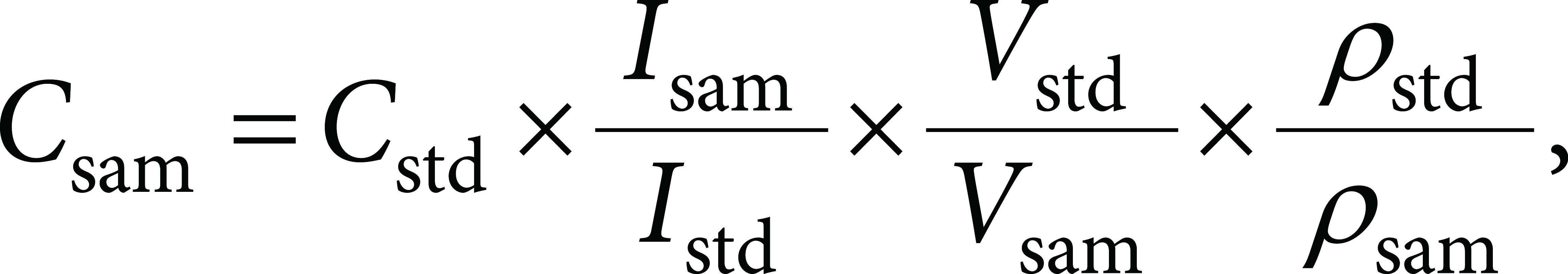
 where *C*_sam_ and *C*_std_ are the concentrations of the sample and standard, respectively; *I*_sam_ and *I*_std_ are the ion counts of the sample and standard, respectively; *V*_sam_ and *V*_std_ are the volumes of laser ablation; and ρ_std_ and ρ_sam_ are the densities of the sample and standard, respectively. Based on a study by Pearce *et al.*, the overall average concentration of La in NIST SRM610 was determined to be 509.2±138.0 μg g^−1^ (ppm). Thus, ρ_std_ was reported as 2.6 g/cm^3^, and ρ_sam_ was defined as 1.0 g/cm^3^.

### Quantitative lanthanum imaging

**Figure 2:**
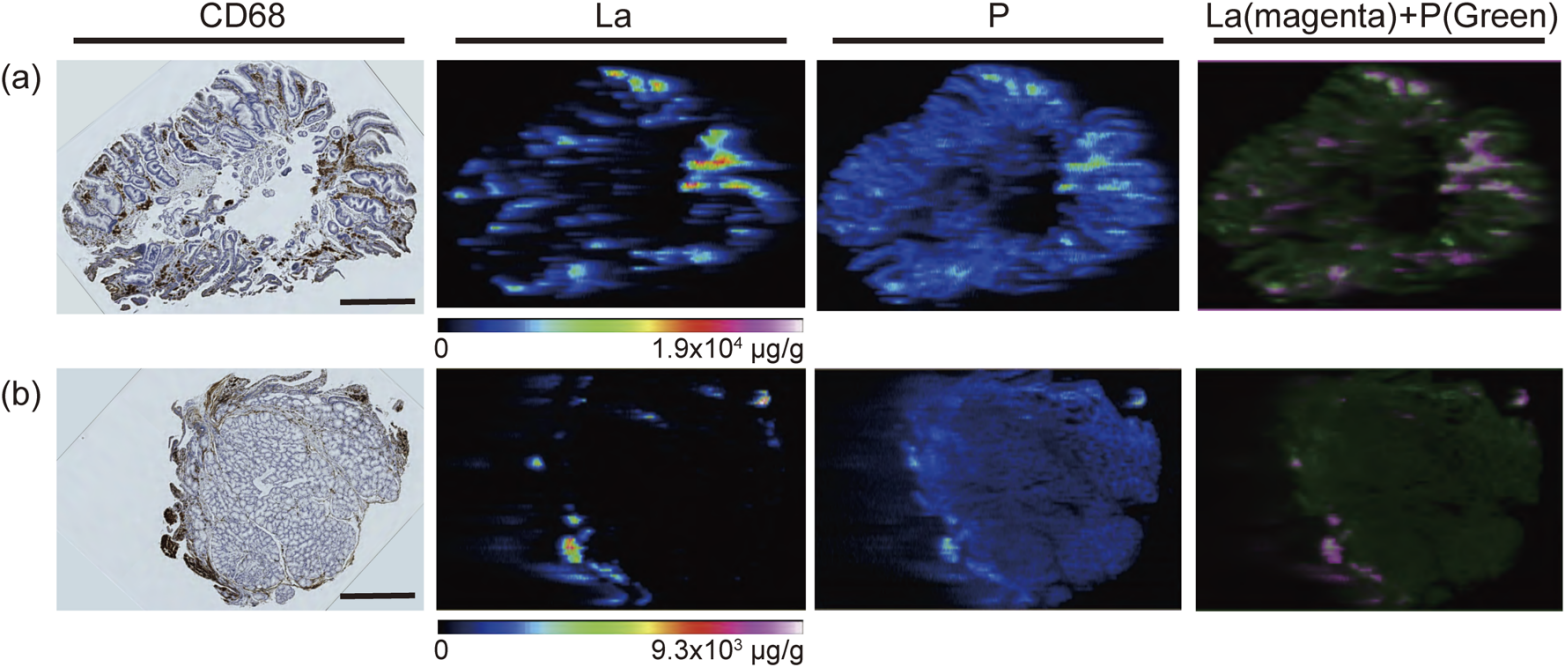
Fig. 2. Comparison of the immunohistochemistry and ^139^La distribution results. Two biopsies were obtained from the same patient and are shown in (a) and (b). In both sections, ^139^La was mainly accumulated at highly CD 68 positive sites. From the quantitation using trace elements in the glass, the maximum concentrations were 1.9×10^4^ and 9.3 ×10^3^ ppm, respectively. Phosphorous images are also provided to confirm tissue morphology (1 μg/g=1 ppm). Scale bars: 500 μm.

Quantitative La imaging was performed using the conversion method described above. Two biopsies (Figs. 2a and 2b) from the same patient were used in this experiment. As shown in Fig. 2a, a region in which La had accumulated was observed in the rightwards section. To visualize the tissue morphology, we also mapped the distribution of phosphorus and provide overlay images of ^139^La (magenta) and ^31^P (green). Comparing the anti-CD68 staining results and the La distribution, the positive regions corresponded to the La accumulated regions. The highest section in Fig. 2a was approximately 10^−2^ ppm/pixel, whereas a hot spot was observed at the bottom left side at 10^−3^ ppm/pixel. Interestingly, some of the anti-CD68 positive sections did not show correspondingly high ^139^La concentrations, especially those shown in Fig. 2b. Based on this result, we conclude that anti-CD68 positive sites were observed even in areas where the concentration of ^139^La was not high because the inflammatory site had spread mainly from the core of the inflammation where large amounts of ^139^La had accumulated.

La imaging is typically performed using optical microscopy, scanning electron microscopy (SEM), and energy-dispersive X-ray spectroscopy (EDS).^14–16)^ However, these studies only report on the qualitative distribution of ^139^La based on macroscopic and microscopic observations. Quantitative data were reported by Namie *et al.* using ICP-MS,^17)^ but the authors did not report on the spatial distribution of ^139^La. In addition, the ^139^La distribution results were not directly compared with histocytes that were positive for CD68 using serial sections. Murakami *et al.* reported on the distribution of ^139^La based on EDS and immunohistochemistry results.^18)^ However, the signal intensity and spatial resolution were insufficient to permit the level of accumulation of ^139^La to be determined. To date, no reports regarding quantitative ^139^La distribution coupled with CD68 immunohistochemistry have appeared. Therefore, our results using LA-ICP-MS represent the first report of the quantitative determination of the distribution of ^139^La coupled with excellent agreement for histocytes.

In addition, as shown in Fig. 2, the areas of the highest accumulation of ^31^P were consistent with the highest accumulation of ^139^La. In previous studies, these distributions were presumed to indicate the presence of lanthanum phosphate.^19)^ We conclude, however, that the observed colocalization is due to the binding of ^139^La with ^31^P which is located on the inner wall of the stomach, with the formation of insoluble lanthanum phosphate.

### High-magnification observations in H&E stained tissues

**Figure 3:**
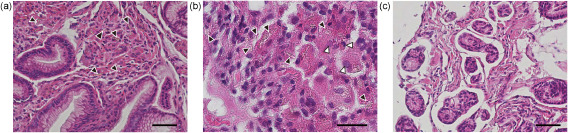
Fig. 3. High magnification H&E staining results for (a, b) a high ^139^La accumulation area and (c) an undetected area, as indicated in Fig. 2a. In (a), brown crystalline debris (black arrowhead) was observed. A higher magnification image in (b) also indicates the presence of clear crystals (black arrowhead). Lymphocytes (white arrowhead) were also observed around the crystals, whereas debris-like material was not observed in the ^139^La undetected area in (c). Scale bars: 50 μm in (a) and (c), 25 μm in (b).

Serial sections were prepared for LA-ICP-MS, anti-CD68 staining, and H&E staining and a representative region with a high accumulation is shown in Figs. 2a and 2b. For this tissue, the same area was observed by H&E staining at high magnification (×40 and ×100; Figs. 3a and 3b). Interestingly, a large amount of crystal-like debris was found in the cytoplasm around the inflammatory regions. In contrast, these morphological materials were not observed near areas of low or zero ^139^La concentrations (Fig. 3c). Therefore, it can be concluded that this debris was derived from ^139^La crystals. The debris was in the form of elongated crystals, similar to those reported by Murakami *et al.* and Makino *et al.*^18,19)^ Inflammation is considered to be induced by these crystal-shaped La formations. In addition to these crystals, leukocytes (mainly lymphocytes) were observed around the debris, supporting our interpretation (Fig. 3b).

## CONCLUSION

We report herein on the quantitative visualization of La using LA-ICP-MS in gastric biopsy samples obtained from a Fosrenol-administrated patient. An inflammation response occurred when La was accumulated at a concentration of a few hundred ppm. This accumulation was in good agreement with the inflammatory response, and a significant amount of debris was observed in these areas, as evidenced by H&E staining. The debris was likely derived from crystals of La. To date, investigations of the distribution of La in human clinical samples using LA-ICP-MS has not been reported. This study provides the first clear correlation between the inflammatory response and the accumulation of La with a high, quantitative spatial resolution.

## Notes

Mass Spectrom (Tokyo) 2020; 9(1): A0086
